# Harm reduction vending machines as safer supply-supporting infrastructure

**DOI:** 10.3389/fpsyt.2026.1955152

**Published:** 2026-09-22

**Authors:** Tessa Rife-Pennington

**Affiliations:** 1 Pharmacy Service, San Francisco Veterans Affairs (VA) Health Care System, San Francisco, CA, United States; 2 School of Pharmacy, University of California, San Francisco, San Francisco, CA, United States; 3 Thomas J. Long School of Pharmacy, University of the Pacific, San Francisco, CA, United States

**Keywords:** drug checking, harm reduction, linkage to care, low-barrier care, overdose prevention, safer supply, syndemic prevention, vending machines

## Abstract

The increasingly unpredictable unregulated drug supply has intensified overdose morbidity and mortality and prompted growing interest in safer regulated supply as an upstream harm reduction strategy. Yet, safer supply programs cannot function effectively as stand-alone interventions. Their reach, acceptability, and equity will depend on surrounding infrastructure that supports people before enrollment, during transitions in substance use or dosing, and alongside ongoing use of unregulated substances. Harm reduction vending machines (HRVMs) are one low-barrier infrastructure model that can support safer supply ecosystems without conflating automated harm reduction supply distribution with regulated drug dispensing. Drawing on implementation experience from a Veteran-serving HRVM program across hospital, outpatient clinic, and permanent supportive housing settings, this article describes how HRVMs can expand access to drug checking supplies, naloxone, safer use materials, wound care and hygiene supplies, HIV and sexually transmitted infection prevention resources, and voluntary care linkage pathways while potentially reducing the need for face-to-face disclosure. Six HRVM infrastructure functions relevant to safer supply implementation are proposed: drug checking access, overdose prevention, safer use and wound care, health and wellness supplies, syndemic prevention, and noncoercive linkage to care. Design and evaluation considerations include privacy protection, product adaptability, co-design with people who use drugs, operational reliability, and the need to distinguish implementation metrics from clinical outcomes. Positioning HRVMs as safer supply-supporting infrastructure may help health systems, community programs, housing providers, and policymakers build more accessible, adaptive, and person-centered harm reduction ecosystems.

## Introduction

The overdose crisis in the United States is driven in large part by an unpredictable unregulated drug supply characterized by high-potency synthetic opioids, variable concentration, and emerging adulterants such as xylazine, nitazenes, and other compounds (1–3). In this environment, people who use drugs (PWUD) often make urgent safety decisions, including whether to test substances, use smaller amounts, avoid using alone, carry naloxone, rely on trusted sellers, or interpret sensory cues, with limited information, time, and access to meaningful alternatives (4–7). Overdose prevention centers, drug checking, naloxone distribution, syringe services, overdose prevention education, medications for opioid use disorder, and related harm reduction interventions are essential components of a comprehensive public health response; however, their population-level impact may be constrained in this volatile drug supply environment (8–11).

Safer regulated supply has therefore emerged as an important upstream harm reduction strategy. Broadly, safer supply refers to legal and regulated access to psychoactive substances or close pharmaceutical alternatives intended to reduce reliance on the unregulated drug market and its associated harms (12). Safer supply approaches are diverse and may include prescribed opioid or stimulant alternatives, supervised or take-home models, pharmacy-linked dispensing, community-governed approaches, or technology-enabled dispensing systems, depending on the legal and clinical context (12, 13). Across these models, the central premise is that preventable harms can be reduced when people have access to substances of known content and potency within a regulated framework (12, 13).

However, safer supply cannot be implemented effectively as a stand-alone intervention. Even when regulated substances are available, people may continue to use unregulated substances before care enrollment, during dose titration, between visits, when prescribed options do not fully meet needs, or when programs are constrained by geography, eligibility criteria, staffing, hours of operation, stigma, or legal restrictions (14–18). Safer supply programs also require surrounding systems that support overdose prevention, drug checking, safer use, wound care, syndemic prevention, health and wellness needs, and voluntary care linkage (8, 17, 18). Without this infrastructure, safer supply risks becoming a narrow clinical or regulatory intervention rather than part of a comprehensive harm reduction ecosystem (13, 14, 18).

This Perspective examines harm reduction vending machines (HRVMs) as low-barrier infrastructure for safer supply ecosystems. HRVMs are automated dispensing systems that distribute harm reduction and related health supplies, such as naloxone, drug checking supplies, safer use materials, condoms, HIV self-tests, hygiene products, and educational resources (19–21). In this model, HRVMs do not dispense regulated psychoactive substances or pharmaceutical alternatives; rather, they can extend access to complementary supports before, during, and alongside safer supply implementation.

## Why safer supply requires complementary infrastructure

Safer supply implementation depends not only on whether regulated substances are available, but also on whether people can reach, use, and remain connected to services in real-world conditions. Programs may be limited by clinic hours, transportation barriers, geography, staffing shortages, eligibility criteria, documentation concerns, stigma, and legal restrictions. People at highest risk of overdose may also be least able or willing to attend scheduled appointments or navigate complex enrollment processes. Low-barrier infrastructure can extend safer supply ecosystems beyond traditional clinical settings (14, 16, 18).

Complementary infrastructure is also needed because safer supply access is likely to be partial, uneven, or transitional in many settings. People may continue to use unregulated substances while awaiting enrollment, during dose titration, between visits, when prescribed options do not fully meet needs, or while using multiple substances. Drug checking supplies, naloxone, safer use equipment, wound care materials, and overdose education therefore remain essential before, during, and alongside safer supply implementation (8, 15, 17).

Finally, infrastructure can support autonomy, trust, equity, and voluntary care linkage. Many PWUD have experienced stigma, surveillance, coercion, or punishment in healthcare, treatment, housing, and criminal-legal systems. Access points that avoid extensive disclosure or mandatory service engagement may reduce barriers and create opportunities for later connection to care (18, 22).

## HRVMs as low-barrier infrastructure

HRVMs are an increasingly visible model for expanding access to harm reduction supplies. Although programs vary in design, HRVMs generally use automated dispensing technology to make supplies available outside traditional service encounters. Machines may be placed in community settings, hospitals, outpatient clinics, supportive housing, shelters, correctional reentry settings, or other locations where people already live, receive services, or seek care. Several features make HRVMs relevant to safer supply ecosystems: flexible access when staff are unavailable, reduced need for face-to-face disclosure of stigmatized needs, adaptable product categories. They can also generate implementation data, such as product uptake, site-level use, stockouts, and repeat use patterns, that may help programs monitor reach and adjust operations (19–21).

The value of HRVMs is therefore not simply that they dispense products; it is that they create a durable, adaptable access pathway. In a safer supply ecosystem, this pathway can support people who are not yet enrolled in safer supply, people who continue to use unregulated substances, and people who need adjacent prevention and care resources. However, this pathway is incomplete on its own. HRVMs cannot provide clinical assessment, peer support, crisis response, wound evaluation, or relationship-based care, and evidence for clinical and cost effectiveness remains limited. Their safer supply-supporting role depends on integration with staffed services, reliable stocking and maintenance, accessible design, data governance, participant and community input, and evaluation of acceptability, cost, and sustainability.

**Figure 1** presents a conceptual model of HRVMs as safer supply-supporting infrastructure. Safer regulated supply is positioned as the central upstream intervention, surrounded by six HRVM-supported infrastructure functions: syndemic prevention, noncoercive care linkage, drug checking access, health and wellness supplies, safer use and wound care, and overdose prevention. These functions are grounded in implementation principles, including low-barrier access, privacy protection, co-design with PWUD, adaptable product mix, and nonpunitive operations.

**Figure 1:**
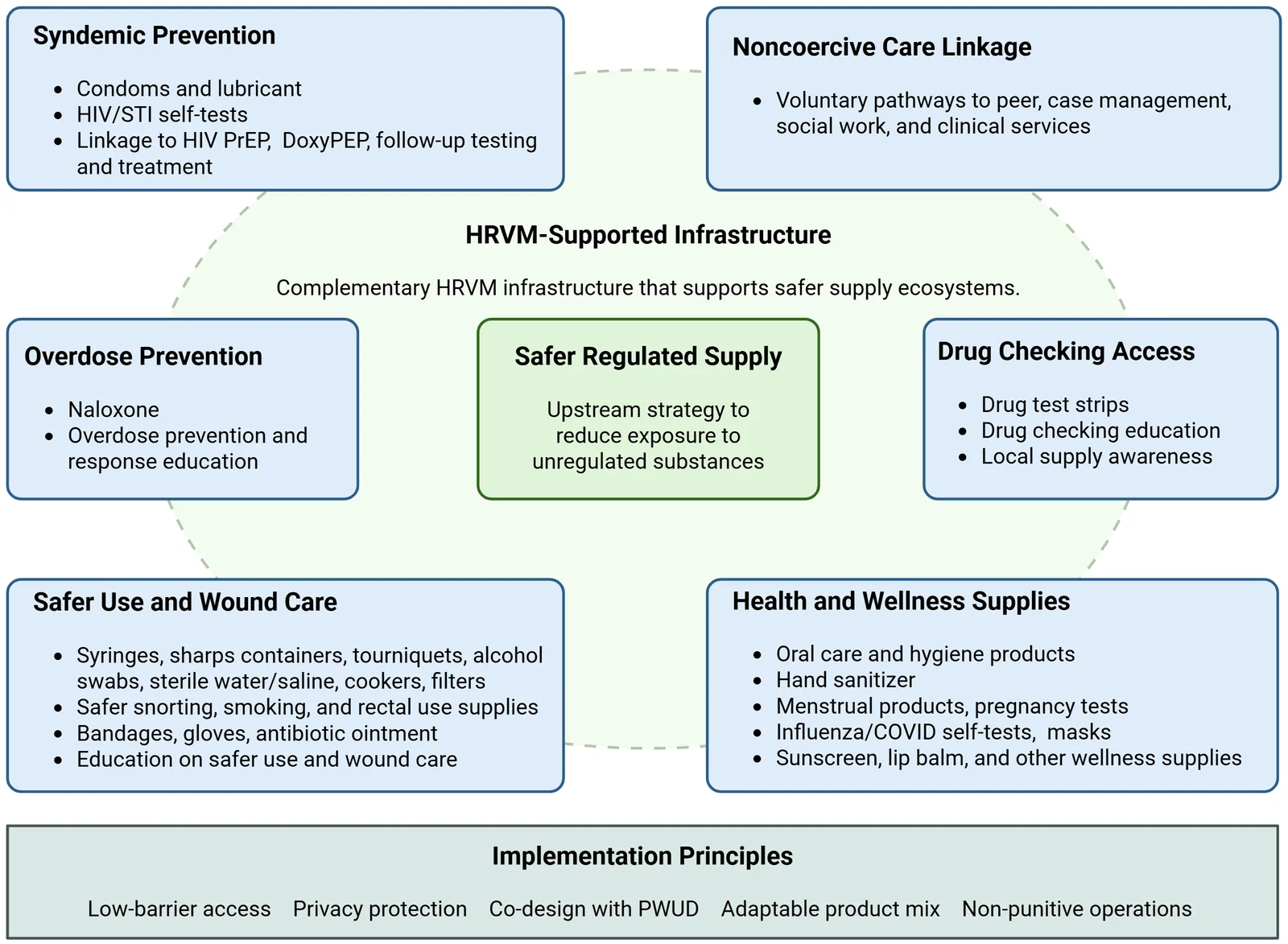
HRVMs as safer supply-supporting infrastructure. Safer regulated supply is positioned as an upstream strategy to reduce exposure to unregulated substances. HRVMs do not replace safer regulated supply or dispense regulated substances in this model. Instead, they provide complementary infrastructure that can support safer supply ecosystems through six functions: syndemic prevention, noncoercive care linkage, drug checking access, health and wellness supplies, safer use and wound care, and overdose prevention. These functions are grounded in implementation principles including low-barrier access, privacy protection, co-design with people who use drugs, adaptable product mix, and nonpunitive operations. HRVM, harm reduction vending machine; STI, sexually transmitted infection; PWUD, people who use drugs; DoxyPEP, doxycycline post-exposure prophylaxis. Created in BioRender. Rife, T. (2026) https://BioRender.com/295708j.

The framework is conceptual and implementation-oriented, not evidence that HRVMs improve safer supply outcomes. It is adaptable across prescribed, supervised, take-home, pharmacy-linked, community-governed, and technology-enabled safer supply approaches, with specific products and linkage pathways shaped by context. The six functions synthesize published safer supply, HRVM, harm reduction service delivery, and low-barrier care literature. This framework can help programs distinguish supports suited for automated distribution from those requiring staffed modalities, identify design risks, and separate implementation indicators from effectiveness outcomes.

## HRVM-supported infrastructure functions

### Drug checking access

Drug checking can help people identify selected compounds, such as fentanyl or xylazine, in the unregulated supply, but many tools, particularly test strips, do not establish purity, concentration, potency, dose, or overall safety (10). HRVM distribution should therefore include clear education about these limitations. Drug checking remains a downstream intervention because it occurs after a substance has been obtained and does not guarantee access to safer alternatives. Nevertheless, HRVMs can make drug checking supplies more accessible before, during, and alongside safer supply implementation.

### Overdose prevention

Naloxone access is foundational to overdose prevention (9). HRVMs can distribute naloxone directly and pair with education on overdose prevention, recognition, and rescue response. This is particularly important in housing settings, after-hours contexts, and locations where people may use alone or where staff may not be immediately available. Even in safer supply models, overdose prevention remains necessary because people may continue to use unregulated substances, use multiple substances, share substances, experience changes in tolerance, or encounter sedating adulterants.

### Safer use and wound care

Safer use supplies and wound care materials remain central to harm reduction for people who inject, smoke, snort, or use substances rectally. Their inclusion acknowledges that safer supply does not immediately eliminate all unregulated substance use or route-associated risks. Some safer supply models may support changes in injection practices, while others may not fully meet participant needs because of medication, dosing, formulation, or access limitations. HRVMs can help reduce barriers to basic supplies without requiring a clinical visit (8, 23–25).

### Health and wellness supplies

Health and wellness supplies may appear less directly connected to safer supply, but they support dignity, comfort, self-care, and engagement, while broadening the role of HRVMs beyond a narrow drug-use frame (26). PWUD have ordinary health and self-care needs that are often intensified by poverty, homelessness, stigma, and limited access to basic resources (27, 28). Providing wellness supplies through the same low-barrier infrastructure may normalize machine use and create a broader access point (29, 30).

### Syndemic prevention

Overdose risk often overlaps with HIV, STIs, viral hepatitis, unstable housing, mental health conditions, trauma, and criminal-legal exposure in a syndemic context (31–33). HRVMs can support low-barrier sexual health resource distribution and care linkage, including HIV self-testing, condoms, and pathways to confirmatory testing, HIV pre-exposure prophylaxis, and treatment (21, 34). Integrating syndemic prevention into HRVM infrastructure helps avoid siloed intervention design (35).

### Noncoercive linkage to care

HRVMs can support voluntary linkage to care through printed materials, quick response (QR) codes, phone numbers, telehealth pathways, or referral instructions connecting users to peer support, case management, social work, harm reduction teams, addiction treatment, wound care, infectious diseases care, sexual health services, primary care, and social services (36–38). Linkage should be available without making access to supplies contingent on treatment enrollment, abstinence, urine drug testing, appointments, or clinician approval (26).

## Implementation lessons from veteran-serving settings

Experience from a Veteran-serving HRVM program illustrates how the proposed functions can appear in practice within an integrated healthcare system. Beginning August 1, 2023, the program deployed 15 HRVMs across hospital, outpatient clinic, and permanent supportive housing settings, with HRVMs used alongside other distribution modalities such as mailed supplies and in-person outreach (39). Multiple access pathways were important because no single distribution strategy works for everyone (21, 40–42).

Access workflows included a one-time sign-up for a unique bar code and 4-digit pin, introduction to the HRVM locations and products, and additional resource offering. Hospital-based machines may reach Veterans receiving acute, specialty, or urgent care and may support connection after hospitalization or emergency encounters. Clinic-based machines integrate harm reduction access into existing outpatient care settings. Supportive housing-based machines may offer proximity, after-hours access, and an option for more private access, depending on program design, in locations where overdose risk, isolation, and unmet health needs may be concentrated (39).

Stocking decisions were informed by program goals, local feasibility, product utilization, legal and institutional constraints, and feedback from Veterans, staff, and implementation partners. Supplies included overdose prevention tools, drug checking supplies, safer use and wound care materials, sexual health and HIV/STI prevention resources, hygiene and wellness items, and care linkage information. Stocking decisions were adapted over time in response to emerging local needs, including increased demand for specific drug checking supplies, expanded hygiene products, and non-prescription naloxone (39).

Implementation measures such as test strips dispensed, sign-ups, machine use, and distribution modality can describe utilization, uptake, and how access is delivered, but should be interpreted cautiously. Reach requires a defined target population or denominator, and operational feasibility requires additional measures such as stockouts, uptime, maintenance needs, acceptability, cost, and sustainability. These measures do not establish clinical effectiveness, behavior change, overdose reduction, infection prevention, or reduced acute care utilization.

Veteran-serving settings also highlight the importance of privacy and stigma reduction. Veterans may avoid harm reduction supplies if they fear judgment, documentation, consequences for pain care or mental health treatment, or negative interactions with staff. Automated access does not eliminate these concerns, but it may reduce some barriers by allowing people to obtain supplies with less direct disclosure. At the same time, HRVMs must be implemented carefully to avoid creating new forms of surveillance or mistrust. Data collection should be minimized, transparent, and limited to what is necessary for program operations, safety, and evaluation.

## Design and evaluation considerations

Because HRVMs can support safer supply ecosystems only when they reliably expand access without creating new barriers, design and evaluation should focus on the conditions that allow machines to function as supportive infrastructure. Programs implementing HRVMs should treat low-barrier access, privacy, nonpunitive operations, and equity as measurable design conditions rather than inherent features of HRVMs. Access workflows should be simple and respectful, and access should not depend on abstinence, treatment engagement, urine drug testing, appointments, or clinician permission. Low-barrier access refers to the ability to obtain supplies with minimal documentation, flexible timing, clear instructions, and no mandatory clinical encounter. Nonpunitive operations means that HRVM use should not trigger sanctions, loss of services, restrictions on pain care or mental health treatment, housing consequences, or criminal-legal exposure.

Design choices can also create new barriers. Registration requirements, authentication codes, phone or internet requirements, electronic logs, institutional cameras, visible placement, stigmatizing signage, limited language options, low literacy accessibility, visual or cognitive accessibility barriers, mobility barriers, stockouts, machine failures, and community or institutional opposition may reduce reach, limit equity, or increase mistrust. Mitigation strategies include transparent data practices, multilingual plain-language instructions, physically accessible and easy to find placement, routine monitoring of stockouts, machine uptime, and maintenance needs, and mechanisms for PWUD and community feedback. Whenever possible, aggregate implementation data should support stocking, maintenance, and evaluation without unnecessary individual-level surveillance.

Co-design with PWUD should guide machine placement, product selection, access rules, educational language, linkage pathways, and evaluation priorities. Programs should also plan for operational reliability, including procurement, stocking workflows, maintenance, inventory monitoring, troubleshooting, and staff accountability. Because drug markets, infectious disease risks, housing needs, and participant preferences change over time, HRVMs should be treated as adaptable infrastructure rather than static supply cabinets.

Evaluation should be structured around this safer supply-supporting pathway by distinguishing HRVM inputs, access functions, implementation outcomes, and clinical and population outcomes. Inputs include HRVMs, stocking workflows, supplies, educational materials, linkage pathways, privacy safeguards, and operational staffing. Implementation outcomes include reach, adoption, feasibility, acceptability, fidelity, cost, sustainability, product uptake, site-level use, stockouts, after-hours access, reduced access burden, and distribution modality (43). Hypothesized safer supply, clinical, and population outcomes, such as voluntary referral, safer supply initiation or retention, drug checking use, naloxone readiness, safer use practices, wound care engagement, infectious disease prevention, linkage to care, overdose outcomes, acute care utilization, or mortality, require additional methods such as mixed methods evaluation, participant surveys or interviews, administrative data linkage, or prospective study designs. Until such data are available, claims about HRVM effectiveness should be appropriately limited.

## Discussion

Safer supply responds upstream to the unpredictable drug supply, but implementation depends on surrounding infrastructure. HRVMs offer one practical model for extending access to the supplies and linkage pathways that remain necessary before, during, and alongside safer supply implementation. In turn, HRVMs can help create a more accessible and person-centered harm reduction ecosystem.

The contribution of HRVMs is infrastructural and modality-specific. Automated distribution may add value when barriers involve timing, privacy, geography, transportation, repeated access to routine supplies, or limited staffing. HRVMs may be less appropriate when people need relationship-based support, complex decision-making, wound assessment, crisis response, language assistance, disability accommodation, or active care navigation. HRVMs should therefore supplement, not replace, peer-led outreach, clinical care, supportive housing services, community harm reduction organizations, mobile services, or mail-based distribution.

This Perspective has limitations. The role of HRVMs as safer supply-supporting infrastructure remains underdeveloped. Existing HRVM evidence is still emerging, and implementation models vary across settings. The Veteran-serving example described provides practical implementation experience but should not be interpreted as a clinical outcomes evaluation. Available distribution metrics can describe utilization and uptake, but they cannot establish reach without a defined target population or a denominator, nor can they demonstrate reductions in overdose, infection, or healthcare utilization. Legal and institutional constraints may limit which products can be stocked, especially syringes, safer smoking supplies, drug checking materials, and self-testing tools. The framework was not developed through a formal consensus process or validated framework-development method. Although Veterans and implementation partners informed aspects of the HRVM program and related Veteran-facing materials, future work should refine and test this model with PWUD, safer supply participants, peer-led organizations, and community-based programs.

Despite these limitations, HRVMs offer a useful framework for thinking beyond safer supply as a singular intervention. Jurisdictions and health systems considering safer supply models should also plan for the surrounding infrastructure required to support safety, autonomy, and engagement. Funding should include not only regulated substances and clinical staffing, but also harm reduction supplies, machine maintenance, stocking workflows, privacy protections, evaluation, and community or participant governance. Policymakers should provide legal clarity for distribution of harm reduction supplies and support low-barrier models that do not punish ongoing substance use.

Safer supply implementation will be strongest when it is embedded in comprehensive harm reduction ecosystems. HRVMs are one tool for building those ecosystems. By positioning HRVMs as safer supply-supporting infrastructure rather than safer supply itself, health systems, housing providers, community programs, and policymakers can better design interventions that are accessible, adaptive, and grounded in the real-world needs of PWUD.

## Data Availability

The original contributions presented in the study are included in the article/supplementary material. Further inquiries can be directed to the corresponding author.
